# Foliar resistance to *Rhizoctonia solani* in Arabidopsis is compromised by simultaneous loss of ethylene, jasmonate and PEN2 mediated defense pathways

**DOI:** 10.1038/s41598-021-81858-5

**Published:** 2021-01-28

**Authors:** Brendan N. Kidd, Rhonda Foley, Karam B. Singh, Jonathan P. Anderson

**Affiliations:** 1 Centre for Environment and Life Sciences, CSIRO Agriculture and Food, Floreat, WA Australia; 2 Australian Reseach Council Centre of Excellence in Plant Energy Biology, School of Molecular Sciences, The University of Western Australia, Crawley, WA Australia; 3 Department of Environment and Agriculture, Centre for Crop and Disease Management, Curtin University, Bentley, WA Australia; 4 The UWA Institute of Agriculture, The University of Western Australia, Crawley, WA Australia

**Keywords:** Plant hormones, Plant immunity, Plant signalling, Plant stress responses

## Abstract

*Rhizoctonia solani* causes damaging yield losses on most major food crops. *R. solani* isolates belonging to anastomosis group 8 (AG8) are soil-borne, root-infecting pathogens with a broad host range. AG8 isolates can cause disease on wheat, canola and legumes*,* however *Arabidopsis thaliana* is heretofore thought to possess non-host resistance as *A. thaliana* ecotypes, including the reference strain Col-0, are resistant to AG8 infection*.* Using a mitochondria-targeted redox sensor (mt-roGFP2) and cell death staining, we demonstrate that both AG8 and a host isolate (AG2-1) of *R. solani* are able to infect *A. thaliana* roots. Above ground tissue of *A. thaliana* was found to be resistant to AG8 but not AG2. Genetic analysis revealed that ethylene, jasmonate and *PENETRATION2-*mediated defense pathways work together to provide resistance to AG8 in the leaves which subsequently enable tolerance of root infections. Overall, we demonstrate a significant difference in defense capabilities of above and below ground tissue in providing resistance to *R. solani* AG8 in Arabidopsis.

## Introduction

Plants have evolved complex detection and response systems to protect against abiotic and biotic stresses. Infection by fungal pathogens leads to the activation of rapid basal defense responses at the cell wall, known as pre-invasion defenses, followed by activation of a defense response that is tuned specifically towards the invading pathogen, known as post-invasion defenses^1,2^.

The plant hormones jasmonic acid (JA), ethylene (ET) and salicylic acid (SA), are predominantly associated with plant defense responses^3,4^. However, as hormone mediated defense responses are responsive to the regulatory network of the plant, other plant hormones and signalling pathways, including metabolic pathways are also involved in regulating plant defense^3–5^. The SA-associated defense pathway is classically associated with defense against biotrophic pathogens that require plant cells to be alive to obtain nutrients, while the JA/ET-associated defenses are typically most effective against necrotrophs, pathogens which kill plant cells to obtain nutrients^6^. Significant progress has been made towards identifying the genetic components underlying resistance against adapted and non-adapted biotrophic fungi using the model plant *Arabidopsis thaliana*^1,2^. For instance, non-adapted fungi are typically repelled at the cell wall through the formation of cell wall papillae and penetration associated defenses mediated by the *PENETRATION* (*PEN*) genes in combination or separately to other downstream defense responses^1^.

However, knowledge of defense against adapted and non-adapted necrotrophic fungi is still limited, despite the significant, on-going losses they cause to a wide range of crops. Resistance against necrotrophs, particularly broad host range necrotrophs, is typically defined by multiple quantitative components, including the production of phytoalexins, reactive oxygen species, and hormone regulated pathogenesis genes and is therefore difficult to unravel using genetic analysis^6–11^.

*Rhizoctonia solani* is a species complex containing several soil-borne necrotrophic fungi known for their ability to cause predominantly root and stem rots, but can also cause leaf diseases on rice and tobacco^12,13^. Despite the *R. solani* species complex containing economically important pathogens, robust R-gene mediated genetic resistance similar to that achieved against biotrophic pathogens, has not been identified for most *R. solani* hosts including rice, wheat, potato and soybean^13^. Individual isolates of *R. solani* are grouped into anastomosis groups based on their ability to fuse hyphae and exchange nuclei^12^. Due to the low occurrence of sexual spore formation, the anastomosis groups are essentially reproductively isolated groupings within the species complex and this is reflected in the large divergence between the genome sequence of isolates from different anastomosis groups^14,15^. Some isolates, such as those belonging to Anastomosis group 8 (AG8) have been shown to have a broad host range; infecting cereals, legumes and canola, whereas other isolates, such as those belonging to AG2-1, have a narrow host range and specialise on crucifers such as canola and Arabidopsis^16,17^. Screening 40 Arabidopsis ecotypes demonstrated that all were resistant to AG8 infection^11^. Conversely, all 40 Arabidopsis ecotypes were highly susceptible to AG2-1^11^.

We have previously used Arabidopsis as a model pathosystem to study how plants defend against necrotrophic pathogens such as *R. solani*^11,18^. Two Arabidopsis mutants have been identified to be susceptible to AG8 and both are associated with reactive oxygen species (ROS) production. The first mutant identified was *dsr1* (*disrupted stress responses1*), which is an EMS mutant in the mitochondrial protein succinate dehydrogenase (SDH) that was found to have reduced mitochondria-derived ROS production^19,20^. The second mutant genotype identified to be susceptible to AG8 was a double mutant in the membrane localised NADPH oxidase genes *RESPIRATORY BURST OXIDASE HOMOLOG D* and *RESPIRATORY BURST OXIDASE HOMOLOG F* (*rbohd rbohf*)^11^. In addition, redox and ROS associated genes were found to be induced in response to AG8 infection using microarray analyses of Arabidopsis and wheat^11,21^, supporting a role for mitochondria and reactive oxygen production in providing resistance to *R. solani*. Mitochondria have been found to localise to penetration sites during infection of Arabidopsis leaves with non-host powdery mildew pathogens^22^. Using the mitochondria localised redox sensor *mt-roGFP2*^23^, mitochondria underneath powdery mildew penetration sites became immobilized and demonstrated increased oxidation in response to infection^22^.

In this report we have used the mitochondria-localised redox reporter, *mt-roGFP2,* and confocal microscopy to analyse root infection with *R. solani* AG8 and AG2-1. Surprisingly, we found that both AG8 and AG2-1 isolates were able to infect Arabidopsis roots and caused cell death at the infection site and surrounding tissue. In addition to infecting roots, the host-adapted AG2-1 isolate caused extensive necrosis on foliar tissue while AG8 was unable to infect wild type Arabidopsis foliar tissue. Through mutant analysis we reveal that defense against AG8 in the foliar tissues requires ethylene, jasmonate and *PENETRATION2* associated defense responses. Overall, we demonstrate an important role for foliar tissue in defense against a necrotrophic root pathogen that has a broad host range on both monocot and dicot crop species. Further investigation of the molecular basis behind organ specific resistance to *R. solani* AG8 in Arabidopsis may provide strategies for improving resistance to AG8 in wheat, canola and legume crops.

## Results

### AG8 and AG2-1 infection causes spreading cell death in Arabidopsis roots

Mitochondrial oxidation has been shown to play a role in defense of Arabidopsis against non-host pathogens^22^ and a mutant in the mitochondrial *SDH* gene was found to be susceptible to the *R. solani* isolate AG8^20^. Therefore, we hypothesized that mitochondria may play a role in defense against AG8 to which Arabidopsis appears to exhibit a non-host-like resistance response*.* To explore whether mitochondria have a role in protecting against *R. solani* we chose to examine the redox status of mitochondria in response to infection with the AG8 isolate as well as the crucifer-specialist isolate, AG2-1, using the mitochondria-targeted redox sensitive GFP probe, *mt-roGFP2*^23^. To confirm the transgenic *mt-roGFP2* Arabidopsis line was functioning appropriately we treated *mt-roGFP2* seedlings with 10 mM H_2_O_2_ and 10 mM dithiothreitol (DTT) and measured fluorescence in root epidermal cells using confocal microscopy. Hydrogen peroxide (H_2_O_2_) treatment results in the oxidation of cysteine bonds in *mt-roGFP2* and a change in excitation preference from 488 to 405 nm^23,24^. A 10 min treatment with H_2_O_2_ effectively oxidised the *mt-roGFP2* probe producing a 405 nm/488 nm fluorescence ratio approximately four times higher than water treated control roots (Fig. S1A-D). Further treatment of the same root with DTT restored the *mt-roGFP2* probe to a ratio equivalent to the control, confirming that the fluorescent probe was functioning as expected (Fig. S1C-D)^23,25^.

**Figure 1:**
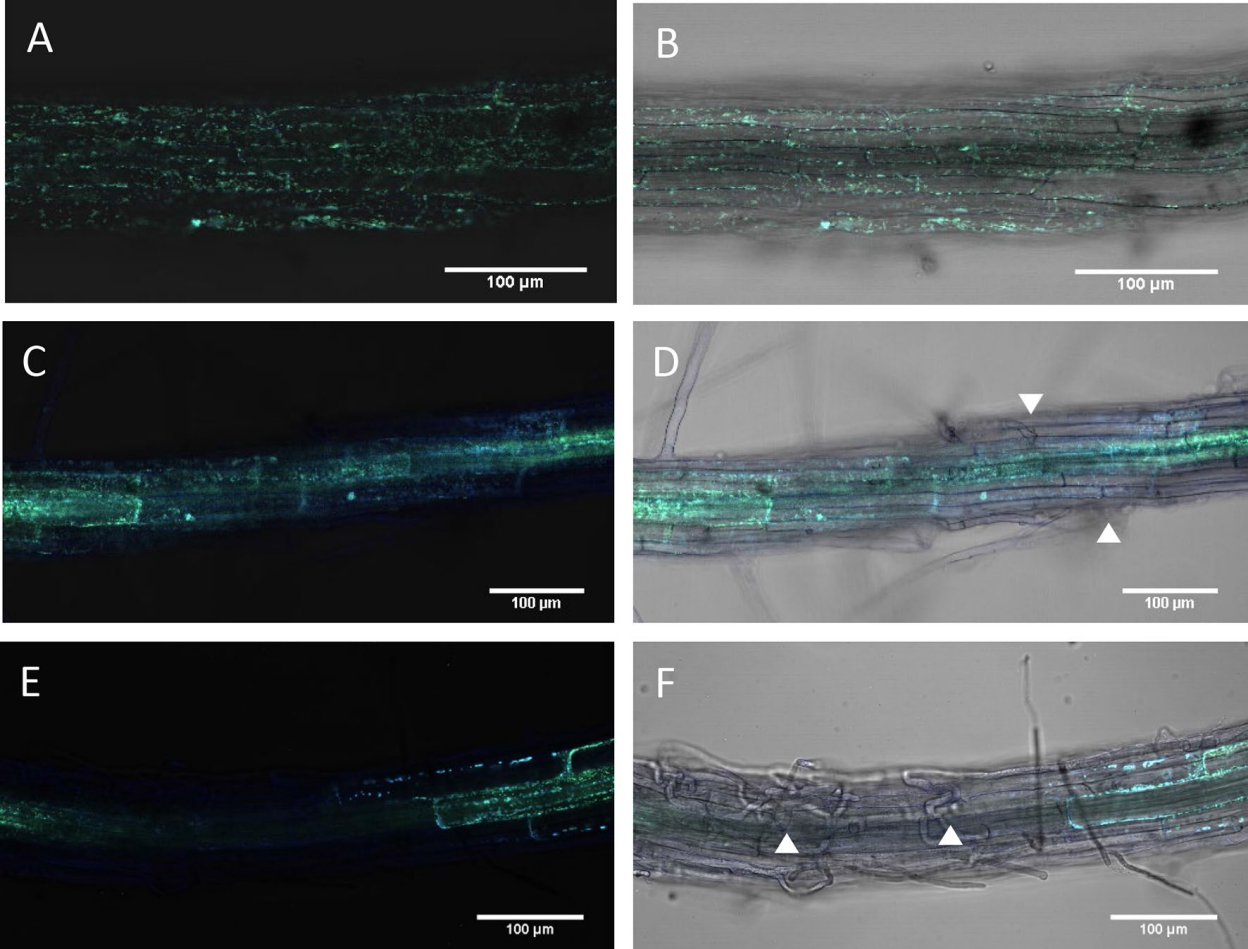
(**A**,**B**) Mock (**C**,**D**) AG2-1 and (**E**,**F**) AG8 infected *mt-roGFP2* plants showing oxidation and loss of GFP expression in the epidermal and cortical cells after *R. solani* infection. Ratiometric confocal images were collected 48 h after infection or mock treatment and represent the ratio of fluorescence emission from excitation with the 405 nm (blue) and 488 nm (green) laser lines. Arrow heads represent infection sites. Scale bar represents 100 µm in all images. A minimum of 20 plants were imaged for each treatment and representative images were chosen.

**Figure 2:**
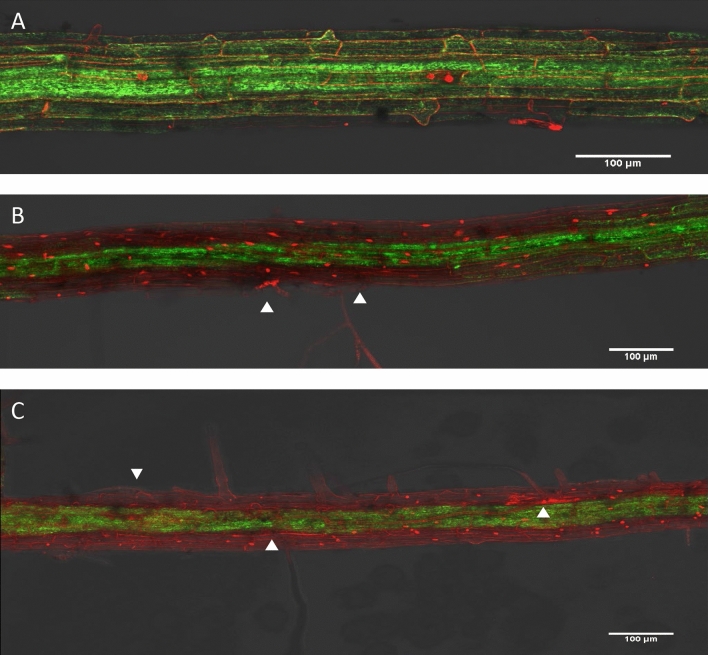
(**A**) Mock, (**B**) AG8 and (**C**) AG2-1 infected *mt-roGFP2* plants showing loss of GFP expression in the epidermal and cortical cells after *R. solani* infection. Images were collected 48 h after infection or mock treatment to capture fluorescence emission from GFP and propidium iodide (PI). Arrow heads represent location of infection hyphae. Scale bar represents 100 µm in all images. A minimum of 10 plants were imaged for each treatment and representative images were chosen.

To examine redox changes in response to infection with the root pathogen *R. solani*, agar grown *mt-roGFP2* plants were transferred into vermiculite pots with or without *R. solani* inoculum before gently removing seedlings for imaging on a confocal microscope*.* This infection system enabled us to examine *R. solani* infection more clearly compared to previous agar-based inoculations where extensive *R. solani* hyphal growth limits observation of infection sites due to the abundance of hyphae on the roots^18^. Interestingly, we observed loss of GFP expression in both AG8 and AG2 infected root epidermal and cortical cells while mock-infected plants did not show a loss of GFP expression under the same growth conditions (Fig. 1A-F). Root cells adjacent to the infection site of AG8 and AG2-1, showed increased fluorescence from the oxidised form of mt-roGFP2 or lost GFP expression entirely (Fig. 1C-F). We noticed that *R. solani* AG8 and AG2-1 infection of Arabidopsis roots could occur through single hyphal tip-mediated infection (Fig. 1D) or through multiple, branched hyphae ramifying through the root tissue (Fig. 1F) with AG8 and AG2-1 adopting both infection styles in order to reach the stele (Supplementary Figs. S2-S3). Quantification of the redox status of individual penetrated cells proved problematic due to several factors; (1) the loss of GFP expression at the local infection site as well as multiple cells surrounding the penetrated cell due to the death of those cells, (2) variation in the density of infecting hyphae observed along the same root, and 3) the inability to predict where on the root surface a successful infection would take place over 48 h precluded continuous live imaging collection to capture early penetration events prior to cell death occurring. Nonetheless, infection with either AG8 or AG2-1 resulted in a loss of GFP expression in multiple cells surrounding the infection site suggesting both *R. solani* isolates induce the death of epidermal and cortex cells. We confirmed that the root cells that lost GFP fluorescence were undergoing cell death by staining AG8 and AG2-1 infected *mt-roGFP2* roots with propidium iodide (PI). PI is occluded from the cytoplasm of healthy cells but is able to permeate the plasma membrane and stain nuclei during cell death^26^. Staining with PI showed that despite the striking differences in plant survival following infection with either AG8 or AG2-1, epidermal and cortex cells were indeed undergoing cell death in response to both AG8 and AG2-1 infection, while in uninfected *mt-roGFP2* roots the majority of cells were viable and only a few root hair cells showed nuclear staining of PI (Fig. 2A-C).

### Infection of Arabidopsis roots with *R. solani* AG8 and AG2-1 results in a similar percentage of root death

As root infection with AG8 and AG2-1 appeared to result in cell death locally at the infection site, we hypothesized that AG2-1 might be more successful in colonizing the entire root system and therefore better resourced to colonize the above ground tissue. To examine this hypothesis, we quantified root cell death regions that showed a loss of GFP as a percentage of the total root length of AG8 and AG2-1 infected plants using PI staining over an infection time course. The root measurement software WinRhizo was used to measure PI stained and non-GFP expressing root regions versus GFP expressing roots.

**Figure 3:**
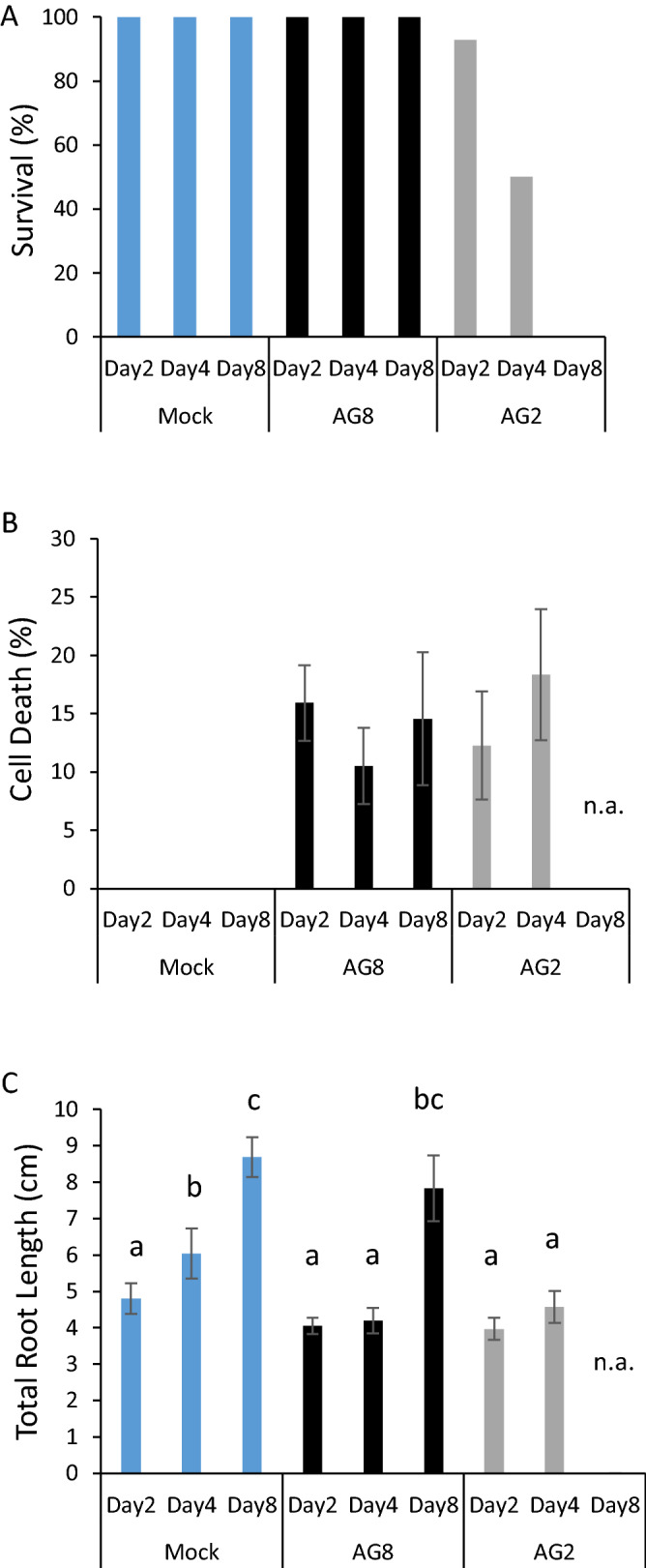
AG8 and AG2-1 isolates cause equivalent levels of cell death on Arabidopsis roots. (**A**) Survival percentage of the ten Arabidopsis plants at each timepoint that were either mock infected or infected with either AG8 or AG2-1. 10 plants were used for each timepoint and treatment for a total of 90 plants. The infection was repeated multiple times and showed similar survival percentages in response to AG8 and AG2. (**B**) The percentage of root cell death compared to the total root length using propidium iodide staining. (**C**) Total root length measurements. The survival, root length and cell death percentage were measured from 10 plants per treatment at each timepoint. Survival of AG2-1 infected plants was also scored at 8 days post infection, however root length and cell death was not assessed (n.a.) as the plants were unable to be isolated from the pots due to disintegration of the above ground tissue. Bars with differing letters represent a significant difference using a one-way ANOVA (*p* < 0.05) and Tukey’s HSD test. Error bars represent standard error.

The percentage of surviving plants for each treatment was calculated per time-point before carefully staining and imaging the entire root system of each plant (Fig. 3A). At the 2 day time-point, AG8 and AG2-1 induced cell death was observed to be 15% and 12% of the total root length, respectively. The average root death percentage of AG8 and AG2-1 infected plants at 4dpi was also similar at less than 20% of the root length, despite approximately 50% of AG2-1 plants showing severe necrosis of the leaves at this stage (Fig. 3A-B, Supplementary Fig S4). AG2-1 plants that were completely necrotic at 4 dpi were measured by carefully teasing out the root systems from vermiculite so as not to bias measurements with only the surviving plants. Leaf tissue with advanced necrosis did not withstand PI staining therefore, only the roots were quantified for cell death. Despite foliar necrosis, GFP expression could still be detected in AG2-1 infected roots, with some of the necrotic plants showing limited cell death in the roots suggesting that colonization of the root system is not a pre-requisite for AG2-1 to infect the aerial tissue of Arabidopsis plants (Supplementary Fig S4A-B). After 4 days of infection, AG2-1 infected foliar tissue had been macerated due to extensive necrosis such that the tissue was severely compromised. Therefore roots from these plants could not be isolated from the vermiculite growth medium and thus it was not possible to measure cell death in these roots reliably.

Infection of Arabidopsis with AG8 and AG2-1 resulted in a significant reduction in total root length at 4 dpi compared to mock inoculated plants (Fig. 3C). Interestingly, the average total root length of AG8 infected plants recovered to a length similar to mock treated plants by the final time-point at 8 dpi suggesting that initial root infection does not prevent Arabidopsis from producing new roots to achieve a total root length similar to uninfected plants (Fig. 3C) suggesting the new roots possess a mechanism for resisting further *R. solani* infection. Overall the root cell death measurements revealed that AG8 and AG2-1 isolates of *R. solani* both cause a similar percentage of cell death in the roots as no statistically significant difference was observed between timepoints or pathogen treatment.

**Figure 4:**
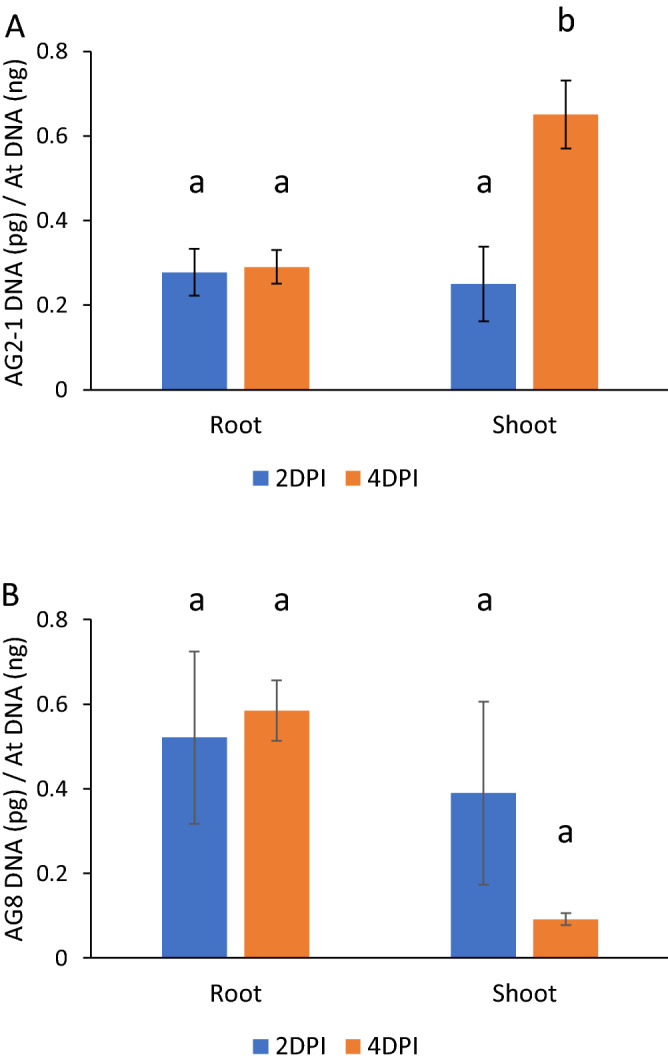
Quantification of (**A**) AG2-1 and (**B**) AG8 biomass in *A. thaliana* Col-0 roots and shoots using qPCR. Col-0 plants were inoculated by planting the roots directly into infected vermiculite. After DNA extraction, pathogen DNA was amplified using *R. solani* ITS primer sequences and Arabidopsis DNA amplified using beta-Tublulin primer sequences. The Y-axis represents relative abundance of *R. solani* DNA (picograms) versus *A. thaliana* DNA (nanograms) determined using a standard curve derived from cultured *R. solani* tissue and *A. thaliana* genomic DNA. Bars with differing letters represent a significant difference (*p* < 0.05) using a one-way ANOVA and Tukey’s HSD test. Six biological replicates of forty plants were used per treatment and timepoint. Error bars represent standard error.

To further quantify AG8 and AG2-1 infection of Arabidopsis, the relative amount of fungal biomass in Arabidopsis root and above ground tissue was analysed using quantitative real-time PCR (qPCR). The amount of in-planta biomass of both AG8 and AG2-1 remained constant in root tissue between the 2 and 4 dpi timepoints (Fig. 4A-B). A statistically significant increase in AG2 biomass was observed in shoot tissue at 4 dpi relative to 2 dpi (Fig. 4A), while no significant change in AG8 abundance was observed between 2 and 4 dpi shoot tissue (Fig. 4B). These results support the notion that Arabidopsis is able to limit AG8 colonisation in the shoots whereas AG2-1 is able to increase biomass in shoot tissue leading to leaf necrosis and plant death.

### Loss of JA, ET and *PEN2* mediated defenses results in a breakdown in leaf resistance to AG8

**Figure 5:**
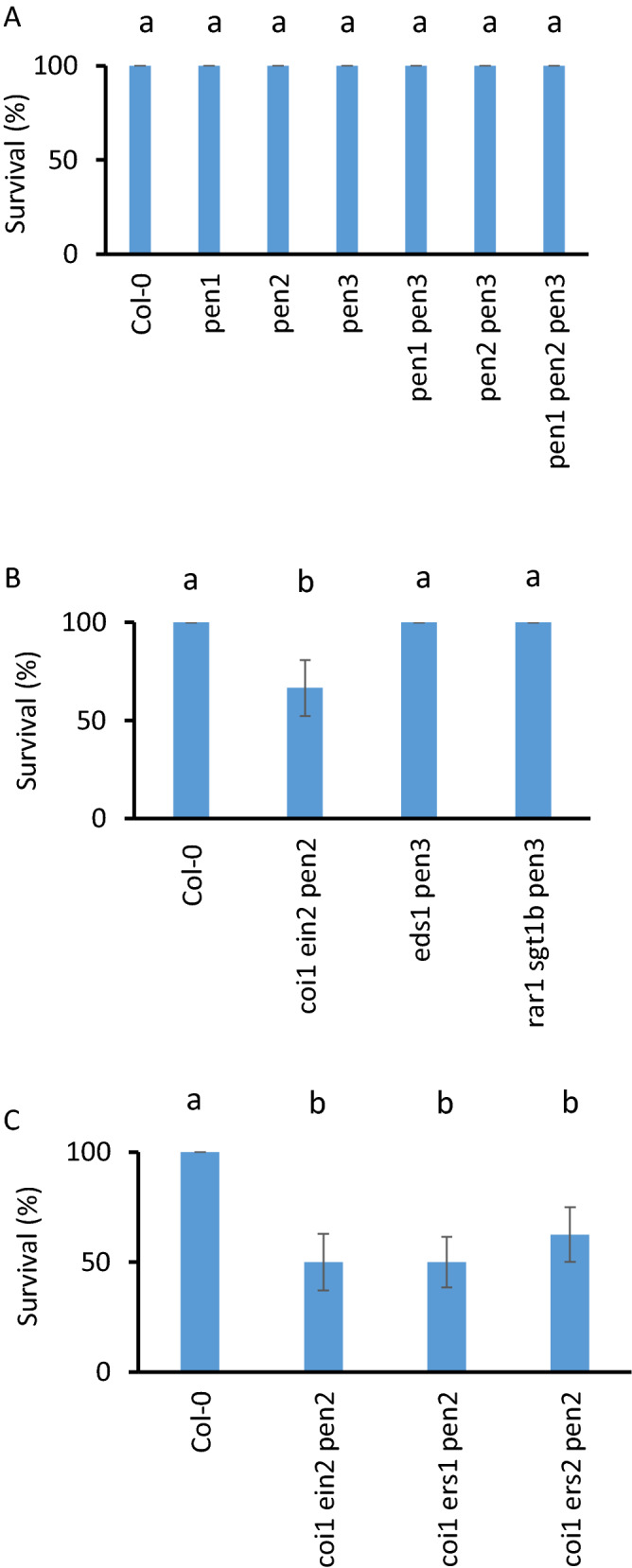
Loss of JA, ET and PEN2 mediated defense pathways compromise resistance to AG8. (**A**) Infection of Col-0, *pen1, pen2, pen3,* single double and triple mutants with AG8. (**B**) Infection of Col-0, *coi1 ein2 pen2*, *eds1 pen3* and *rar1 sgt1b pen3* mutants with AG8. (**C**) Infection of Col-0, *coi1 ein2 pen2*, *coi1 ers1 pen2* and *coi1 ers2 pen2* with AG8. Three replicate infections of agar grown plants were performed by transferring 4 plants per biological replicate into mock or *R. solani* infected pots to produce the data in this figure. Plants were scored for survival at 14 days post infection at which point surviving plants had started to flower. An additional independent experimental repeat of this experiment was also performed which confirmed the result (Supplementary Fig. S5). Letters represents significance (adj. *p* < 0.05) using Fisher’s Exact Test. Error bars represent standard error.

As the percentage of root cell death did not correlate with overall survival, this suggests that successful infection of above ground tissue might be a determining factor in Arabidopsis-*R. solani* interactions. In the Arabidopsis-*R.solani* infection system, foliar infections may be facilitated by the proximity between the infected vermiculite and Arabidopsis leaves. Because AG8 infected plants lack visible symptom development in the above ground tissue, we performed our standard vermiculite infection on previously characterised *PENETRATION* mutants (*pen1, pen2 and pen3;* which play important roles in penetration resistance against leaf infecting non-host pathogens) using our standard root infection method. This revealed that neither the individual, double or triple *pen* mutants affected Arabidopsis survival in response to AG8 infection (Fig. 5A). We also inoculated *pen* mutants that have been previously combined with JA/ET- or R-gene- and SA-associated defense mutants^27–29^. The mutants that were infected included the *coi1 ein2 pen2* mutant which in addition to *pen2* has loss of function mutations in the JA-Ile receptor *CORONATINE INSENSITIVE1* (*COI1*)^30^ as well as a mutation in *ETHYLENE INSENSITIVE2* (*EIN2*)^31,32^, a key regulator in ethylene signal transduction. We also infected the *eds1 pen3* and *rar1 sgt1b pen3* triple mutants. EDS1 together with PAD4, forms a signalling hub which is important for SA signalling and TIR-NB-LRR resistance gene mediated defense^33^, while co-chaperones *SGT1B* and *RAR1* are also associated with resistance gene signalling and facilitate binding with the chaperone HEAT SHOCK PROTEIN90 (HSP90)^34^. Infecting these triple mutants with AG8 identified the *coi1 ein2 pen2* mutant as being susceptible to AG8, while *eds1 pen3* or *rar1 sgt1b pen3* retained resistance (Fig. 5B). We also inoculated *coi1 ers1 pen2* and *coi1 ers2 pen2* triple mutants which have mutations in the ET receptors *ETHYLENE RESPONSE SENSOR1* (*ERS1*) and *ETHYLENE RESPONSE SENSOR2* (*ERS2*) together with the same *coi1-16* and *pen2-4* mutations^29^. The *ers1* and *ers2* mutants confer dominant ethylene insensitivity^35,36^ and the *coi1 ers1 pen2* and *coi1 ers2 pen2* triple mutants were also susceptible to AG8 infection (Fig. 5C). Due to the requirement for cold treatment, we were unable to produce sufficient seeds for all lines due to very low seed production in lines containing the *coi1-16* allele. However, we were able to perform an independent repeat of this experiment with the single, double and triple mutant lines available. A higher level of aggressiveness from the AG8 millet seed infection was observed in this experiment, however similar to previous results, only the *coi1 ein2 pen2* triple mutant showed a significant difference in survival compared to wild type Col-0 (Supplementary Fig. S5). Overall, the results from these experiments demonstrate that combining mutations in JA (*coi1*)*,* ET (*ein2*, *ers1* or *ers2*) and PEN2-mediated (*pen2*) defense*,* leads to a significant decrease in survival to AG8 in Arabidopsis.

**Figure 6:**
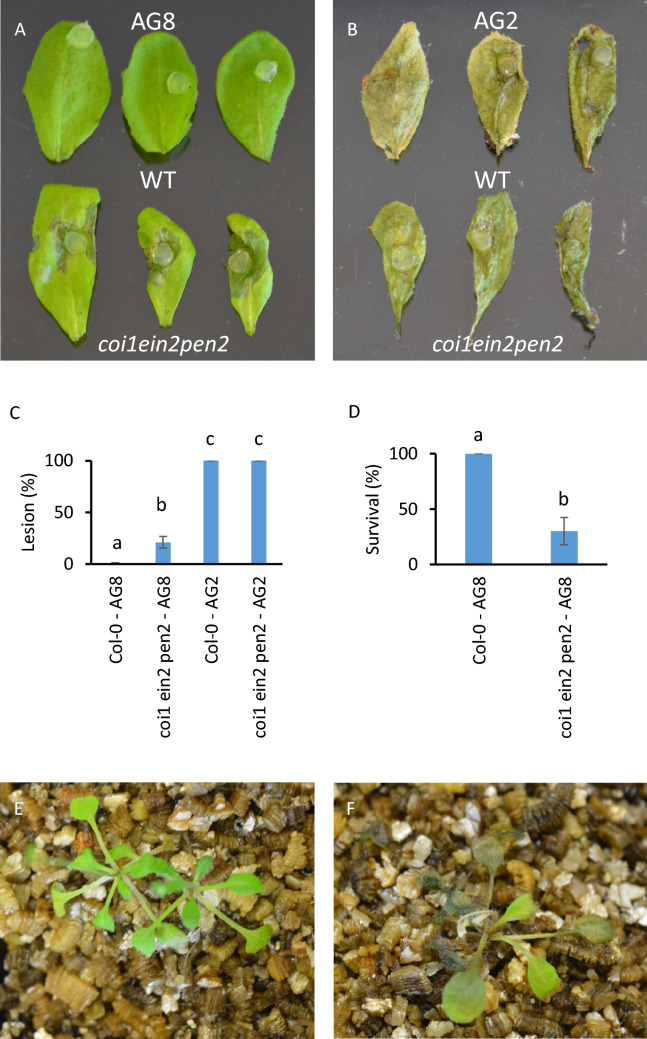
The *coi1 ein2 pen2* mutant is susceptible to leaf infection by AG8. AG8 agar plugs (**A**) or AG2-1 agar plugs (**B**) were added directly to leaves of 6-week old soil grown Col-0 and *coi1 ein2 pen2* plants. The leaves were detached prior to photographing 8 days after infection. Ten leaves were inoculated per genotype for each isolate and the lesion percentage was quantified by measuring the lesion area relative to the total area of the leaf using ImageJ (**C**). The roots of Col-0 and *coi1 ein2 pen2* plants were removed at the hypocotyl junction prior to transplanting in pots of AG8 infected vermiculite. The average survival of five biological replicates is shown (**D**). Representative photographs of Col-0 (**E**) and *coi1 ein2 pen2* (**F**) plants at 1 week after removal of roots and transplanting to infected vermiculite. Error bars represent standard error. Bars with differing letters represent a significant difference (*p* < 0.05) using a one-way ANOVA and Tukey’s HSD test (**C**) or Student’s *T* test (**D**). Error bars represent standard error.

To determine whether the loss of resistance to AG8 in the *coi1 ein2 pen2* triple mutant was due to compromised leaf defense we infected *coi1 ein2 pen2* leaves directly with AG8 and AG2-1 inoculated agar plugs. Infection of Arabidopsis leaves with AG2-1 showed that the WT and *coi1 ein2 pen2* leaves were equally susceptible, while only *coi1 ein2 pen2* but not WT leaves were susceptible to AG8 infection (Fig. 6A-C). As an alternative method to examine foliar resistance, we performed an infection experiment where we removed all root tissue at the hypocotyl junction prior to infection with AG8 in vermiculite. Despite the plants having to regrow their roots through AG8 infected vermiculite in this modified infection system, wild-type plants remained 100% resistant, while *coi1 ein2 pen2* plants were susceptible to AG8 infection similar to previous experiments but with a lower survival percentage (Fig. 6D-F). Overall, these experiments suggest that AG2-1 is highly effective in colonizing and infecting Arabidopsis leaves while AG8 leaf infection is unsuccessful unless a combination of JA, ET and PEN2-mediated defense pathways are compromised.

## Discussion

In this report, confocal microscopy and infection of plant defense mutants was used to observe both cell death responses to *R. solani* infection as well as identify the defense signalling pathways that provide resistance of Arabidopsis to the AG8 isolate of *R. solani*. Previous observations of AG8 and AG2-1 infection of Arabidopsis roots have been performed using an agar plate based assay^18^. However, in an agar based infection system hyphae grow profusely on the agar surface and roots need to be washed thoroughly under running water to remove excess mycelia and observe infection hyphae. Therefore, determining whether hyphae are external or internal proved difficult due to the abundance of hyphae when using agar as the infection medium. In this study, we used a vermiculite infection system with a reduced level of inoculum relative to plate based assays which allowed visualisation of infection sites without extensive washing of the roots and therefore facilitated observations of GFP reporter expression in response to infection. Infection of Arabidopsis roots by AG8 and AG2-1 was observed to occur through direct hyphal tip penetration from a single hyphal tip (Fig. 1D), but also through multiple branched infection hyphae infecting root cells simultaneously (Fig. 1F). During infection, hyphae of *R. solani* are known to branch or overlap through hyphal fusion forming complex structures known as infection cushions^12^. We did not observe infection cushion formation on the root surface however branching of hyphae inside the root cortex after successful infection was observed (Supplementary Fig S2-S3).

Using the *mt-roGFP2* reporter line we observed a loss of GFP fluorescence in epidermal and cortical cells surrounding the infection sites of both AG8 and AG2-1 (Fig. 1–2). In some instances, the cell death response spread well beyond the cells directly infected by *R. solani* but at this stage the mechanism responsible, such as a toxin, elicitor or effector is unknown. Given the difference in survival of Arabidopsis in response to AG8 and AG2-1 infection, the similar degree of root cell death (Fig. 3) and fungal infection of the cortex and stele (Supplementary Fig. S2-S3), from both pathogens was unexpected. The rapid loss of GFP fluorescence in necrotic root cells and difficulty in predicting the location and timing of fungal attempts to penetrate the roots meant the *mt-roGFP2* reporter could not be used for measuring the redox balance of *R. solani* infected cells.

As the above ground phenotype of AG8 infected Arabidopsis resembles a non-host infection without evidence of cell death or necrosis, we aimed to dissect the signalling pathways required for foliar resistance to AG8 through infecting mutants of the *PEN1, PEN2* and *PEN3* genes, which play roles in penetration resistance to several non-adapted pathogens and race specific resistance^28,37^. The *PEN1* gene encodes a syntaxin that are involved in the secretion of exosomes that contribute to the formation of papillae that physically block fungal invasion^38,39^, and is primarily associated with defense against powdery mildews while the *PEN2* and *PEN3* genes are involved in secondary metabolism-based defense against multiple filamentous pathogens^40,41^. *PEN2* encodes a myrosinase that hydrolyzes indole glucosinolates^42^ while *PEN3* encodes an ABC transporter^28^ that secretes camalexin and PEN2-dependent indole glucosinolate metabolic products into the apoplastic space where they act as important induced defense compounds^41,43,44^.

PEN2 was found to be anchored in mitochondria and peroxisomal membranes during *Blumeria graminis* f. sp. *hordei* (Bgh) infection, with the anchored localisation being associated with its role in penetration resistance^22^. In addition to its importance for resistance to the obligate biotroph powdery mildew pathogen *Bgh*, PEN2 derived indole glucosinolate hydrolysis products are thought to be important for quantitative resistance to a necrotrophic pathogen, the leaf-infecting *Botrytis cinerea*^45,46^*,* although other Trp metabolic products may also be involved^44^. In addition, PEN2*-*derived metabolites play only a minor role in resistance against non-adapted isolates of the necrotrophic pathogen *Plectosphaerella cucumerina*^47^. The observation that individual *pen* mutants or the *pen1 pen2 pen3* triple mutant did not affect resistance to AG8, suggests that other defense pathways are able to halt AG8 infection attempts in the leaves. Previous work showed that single mutations in the jasmonate, ethylene or salicylic acid signalling pathways did not influence susceptibility to AG8^11^. However, the identification of susceptibility to AG8 in the *coi1 ein2 pen2*, *coi1 ers1 pen2* and *coi1 ers2 pen2* triple mutants suggests that the defense pathways relying on JA-, ET- and PEN2- are required for the successful defence response in foliar tissue. Transcription factors regulated by the JA- and ET- pathways (E.g. MYC2, MYB34, MYB51, MYB122, ERF6 and others) alter the production of indole glucosinolates in Arabidopsis^41,48,49^ thereby providing a link between the JA- and ET- pathways and PEN2 mediated resistance that is potentially related to the foliar resistance phenotype observed against AG8. Further work is now needed to dissect these pathways with additional mutants to determine their relative contribution to defense against *R. solani* AG8.

Resistance to AG8 was demonstrated to be independent of roots through direct infection experiments on leaves of WT and *coi1 ein2 pen2*. Therefore, the COI1-, EIN2- and PEN2*-*mediated defense pathways successfully resist AG8 infection in above ground tissues. Although Arabidopsis leaf tissue was resistant to AG8, root tissue was surprisingly susceptible. Differences in root and shoot defenses has been observed previously during Arabidopsis infection by the oomycetes, *Phytophthora cinnamomi* and *P. parasitica*^50,51^*.* Similar to what we have observed with AG8 infection of Arabidopsis, *P. cinnamomi* cannot infect leaf tissue but is able to successfully infect root tissue of Arabidopsis without causing visible above ground disease symptoms^50^. Similarly to infection with AG8^11^, inoculation of single JA-, ET- and SA-associated defense mutants did not show a change in resistance to *P. cinnamomi* compared to the wild-type^50^ however, this study did not investigate the impact of double or triple defense mutants on foliar resistance. In addition, the leaves of the Arabidopsis ecotype, Zurich (Zu-1), was shown to be resistant to over 20 different *P. parasitica* isolates however *P. parasitica* could colonize Zu-1 roots^51^. PAMP triggered immune responses as well as hormone signalling and pathogenesis related genes are activated during root infection by filamentous pathogens, however differences in gene expression between roots and shoot infection has been observed^52,53^. Nevertheless, colonization of *R. solani* AG8 seems to be tolerated in Arabidopsis roots substantially more as compared to leaves and therefore future work should examine defense gene expression changes in roots versus foliar tissue after AG8 infection to understand the mechanisms contributing to resistance in the new roots.

A difference in the leaf and root defense responses have also been reported for *Magnaporthe oryzae* isolates infecting Arabidopsis with roots being susceptible to infection, while leaves show penetration resistance^54^, leading to a new interpretation of non-host resistance taking into account organ specificity^55^. Given the above ground survival phenotype, Arabidopsis was previously considered a non-host for *R. solani* AG8^11^. However, the current study reveals that AG8 is capable of infecting Arabidopsis roots but not leaves suggesting Arabidopsis also possesses organ-specific resistance to AG8. It is possible that AG8 contains effectors or toxins necessary to successfully infect Arabidopsis roots but lacks the complement of effectors and toxins to infect the leaves in contrast to the Brassica specialist AG2-1, which was able to infect both leaves and roots of Arabidopsis. A previous study comparing the secretomes of several *R. solani* isolates including AG8, AG1-1A and AG3 identified overlapping but distinct arrays of candidate effectors in the three isolates^56^, suggesting different *R. solani* isolates carry diverse effectors that contribute to the virulence profile of the pathogen. Further elucidation of the effectors and pathogenicity factors employed by *R. solani* AG8 is required to further explore the observed tissue specificity.

The results from this paper demonstrate that above ground symptoms do not always reflect infection success below ground and that a combination of COI1-, EIN2- and PEN2-mediated defense pathways are able to successfully halt AG8 infection in Arabidopsis leaves. The *R. solani* AG8 isolate used in this study causes significant crop losses in many crops including wheat, barley and canola and therefore it is remarkable that Arabidopsis can tolerate root colonization by a pathogen that causes severe root decay and seedling collapse in both monocot and dicot crop species. Improving the defense response of above ground organs during the seedling stage and improving the plants ability to produce new roots during infection may be a useful strategy for genetic improvement of AG8 susceptible crop plants in order to reduce yield loss and economic damage associated with this devastating disease.

## Materials and methods

### Plant growth and mutant lines

All seed lines used in this study were obtained from the Arabidopsis Biological Resource Centre (Supplementary Table S1) with the exception of the *mt-roGFP2* line which was a kind gift from Dr. C.P. Lee^57^, and the *pen1pen2pen3* triple mutant which was a kind gift from Prof. M.X. Andersson^37^. Mutant lines were checked for homozygosity using primers listed in Supplementary Table S2 as well as by phenotyping on ½ strength Murashige and Skoog (MS) media containing 50 µM MeJA and 4 µM ACC for mutants that possess JA- (*coi1*) or ET- (*ein2*) insensitivity. Seeds were surface sterilised using 70% ethanol for 15 min. After sterilization, seeds were suspended in sterile water and washed four times before plating onto ½ MS agar (pH 5.7 with 1% sucrose). After stratification at 4 °C for 2 days, plates were incubated in an upright position in a 22 °C long-day (LD) growth chamber (16 h light at 200 μMol m^-2^ s^-1^) for 14 days before infection with *R. solani*.

### *R. solani* infection experiments

The maintenance and source of *R. solani* strains, AG8 (ZG1-1; WAC10335) and AG2-1: (ZG5: WAC9767), have been described previously^18^. Vermiculite was dispensed into a 30 cell Kwik pot tray and drenched with water prior to being inoculated with four *R. solani*-colonized millet seeds per cell (Garden City Plastics; Australia). The whole tray was then covered with aluminium foil and kept in a plastic bag for 7 days at 22 °C before Arabidopsis planting. Twelve day old seedlings grown on ½ MS media were transplanted to vermiculite. Four seedlings were placed in each cell, keeping the leaves and hypocotyl away from *R. solani-*colonized millet seeds. The roots were covered with fresh vermiculite and watered before returning plants to the 22 °C growth cabinet. Survival of the seedlings was scored 7 to 8 days after infection and re-examined for a change in survival scores every 2 days until the final score was made at 14 days post infection. The data presented in this paper reports the final 14-day survival score (no intermediate phenotypes were observed at this time point). A minimum of twelve plants were inoculated per mutant line (three randomized replicates of four plants per cell). A modified infection was performed by removing roots at the hypotocyl junction with a scalpel before transferring the cut hypocotyl and rosette directly into *R. solani* infected vermiculite. Fisher’s Exact test of Independence, was performed in R version 3.3.3 using the package “rcompanion” and significance set to an adjusted *p* value of less than 0.05 for multiple mutant comparisons, otherwise a two-tailed Student’s T-test or ANOVA was used. Leaf infections were performed by placing AG8 or AG2-1 water agar plugs onto 10, 6-week old Col-0 or *coi1 ein2 pen2* leaves. The plants were lightly sprayed with water and kept in sealed plastic boxes to maintain humidity. The infected leaves were removed from the plant immediately prior to imaging. The area of necrosis relative to the total area of the leaf was measured using ImageJ (available at https://imagej.nih.gov/ij/index.html). The *in-planta* relative fungal biomass of AG8 and AG2-1 was quantified as previously described^11^. Briefly, *R. solani* ITS primer sequences (5′-AGAGTTGGTTGTAGCTGGTCC-3′, 5′-CCGTTGTTGAAACTTAGTATTAGA-3′) were used to amplify both *R. solani* isolates while beta-Tubulin primer sequences (5′-ATCACAGCAATACAGAGCCTTAACC-3′, 5′-GCTGTTGTTATTGCTCCTCCTGCA-3′) were used to amplify Arabidopsis DNA. Forty Arabidopsis Col-0 plants per biological replicate were inoculated with either AG8 and AG2-1 using the standard millet seed root infection in vermiculite. A mock infection was also performed as a negative control. Six replicates of each pathogen treatment were collected at 2 dpi and 4 dpi and DNA extracted for qPCR. Relative abundance of *R. solani* DNA versus *A. thaliana* DNA was determined using a standard curve derived from cultured *R. solani* tissue and negative control *A. thaliana* genomic DNA to quantify *in planta* samples.

## Confocal microscopy of *R. solani* infected seedlings

Ratiometric imaging of 12 day old *mt-roGFP2*-expressing seedlings was performed in 50 mm glass bottomed Mattek dishes (Ted Pella Inc, USA) immersed in water using a Nikon A1Si confocal microscope. The 405- and 488-nm laser lines were used for excitation of the reduced and oxidised forms of *roGFP2* and collected sequentially using an emission band-pass filter of 500–520 nm^57^. A transmitted light image was also captured using the 488 nm laser. Images were acquired using the NIS-Elements software package (version 4.13.01, Build 916) in ‘2Ex 1Em’ mode using a 10x (Nikon CFI Plan Apo DIC L 10 × 0.45 N.A.) or 20 × (Nikon CFI Plan Apo VC 20X 0.75 N.A.) objective with pinhole diameter of 1 airy unit. A laser power ratio of 6:1 (405 nm/488 nm) was kept constant for all images and photomultiplier gain and offset were kept identical between 405 and 488 nm channels. The performance of the *mt-roGFP2* seedlings was validated using treatment with 10 mM H_2_O_2_ for oxidation and 10 mM DTT for reduction of the *mt-roGFP2* probe. Each agar-grown Arabidopsis seedling was mounted in H_2_O in glass bottomed Mattek dishes and imaged to obtain the control measurement. The seedling was then treated with H_2_O_2_ for 10 min and then imaged followed by DTT treatment for 10 min and imaged. This procedure was also independently repeated on separate seedlings. Ratiometric calculations were performed on background corrected maximum plane projections of each z-stack using Redox Ratio Analysis (RRA) software version 1.3 (https://markfricker.org). Scale bar for ratiometric images was added using ImageJ. Mock and *R. solani* infected roots were imaged at one and two days post infection by carefully removing the vermiculite from the roots in water to ensure the roots were not disturbed or damaged, and seedlings transferred to glass bottomed Mattek dishes by gently lifting the cotyledon leaves. Over 10 independent infection experiments were examined and between 2 and 8 plants per AG8, AG2-1 or mock treatment were examined at each experimental sitting at the confocal for each timepoint. Aniline blue staining for images in Figs. S1 and S2 was performed by staining *R. solani* infected Arabidopsis roots, 2 days after infection for 2 min in 0.1% Aniline blue in a 30% Lacto-glycerol (30% Lactic acid, 30% Glycerol) solution. Roots were rinsed in 30% glycerol before imaging using a 40x (Nikon Plan Fluor 40 × Oil DIC) objective. Images were converted to an .AVI file using ImageJ.

### Cell death measurements

Mock and infected *mt-roGFP2* plants were carefully harvested from vermiculite to avoid breaking any roots at two, four, and 8 days post infection and stained with 1 μg/ml propidium iodide (PI) (Thermo-Fisher, Australia) for 1 min, carefully transferred into a dish of distilled water to remove excess PI staining and then placed on a glass slide with a rectangular coverslip. Seedlings were imaged using a Leica M205FA stereo microscope and a Leica DMC4500 colour camera and a long-pass GFP filter (Excitation 480 nm, Emission 510 nm LP) (Leica, Australia) in a 5 × 11 Tilescan at 25 × magnification covering the entire root length (55.5 mm × 22.3 mm) in the X and Y dimensions. Seedlings were positioned such that the hypocotyl-root junction was aligned to the left hand edge of the coverslip and tilescan imaging and assembly of images was then performed automatically using Leica Application Suite X (version 1.90.1374) (Leica, Australia). The colour images were then inverted using Irfan View version 4.38 (available at http://www.irfanview.com) and copied onto A4 transparency paper (Nobo, Australia) and scanned using an EPSON V700 flatbed scanner (Epson, Australia) to calculate whole root lengths. The colour contrast between GFP and PI staining was used to identify root cell death regions. Regions that showed only PI fluorescence and no GFP fluorescence were also transferred onto transparent film (Nobo, Australia) and scanned. The total root lengths and the length of cell death root regions were measured using WinRhizo 2008a Pro^58^. Preliminary experiments were performed using mock-treated and *R. solani*-treated infected plants to ensure that the measurements made by WinRhizo accurately measured the true root length of the root regions. To validate the measurements from WinRhizo the images were magnified and the percentage of cell death regions calculated manually. Cell death as a percentage of total root length was measured for ten AG8, AG2-1 and control treated plants per time-point. AG2-1 infected plants could not be measured at the 8 day timepoint as the above ground tissue had been degraded for multiple days by this timepoint and therefore root material was not able to be collected.

## Supplementary Information

Supplementary Information.

Supplementary Video.

Supplementary Video.
