# Supplementary material for: Foliar resistance to Rhizoctonia solani in Arabidopsis is compromised by simultaneous loss of ethylene, jasmonate and PEN2 mediated defense pathways
Source: Sci Rep. 2021 Jan 28;11:2546. doi: 10.1038/s41598-021-81858-5 (PMC7843637; doi:10.1038/s41598-021-81858-5)
Supplement: Supplementary file 1 — Supplementary Information. [file 41598_2021_81858_MOESM1_ESM.pdf]

Foliar resistance to *Rhizoctonia solani* in Arabidopsis is compromised by simultaneous loss of ethylene, jasmonate and PEN2 mediated defense pathways

Brendan N. Kidd, Rhonda Foley, Karam B. Singh, Jonathan P. Anderson

Supplementary information

Supplementary Figure S1. Validation of the *mt-roGFP2* line in *A. thaliana* root epidermal cells. (A) Confocal image of an untreated control *mt-roGFP2* root showing an overlay of the ratio of oxidised and reduced *mt-roGFP2* fluorescence collected after 405nm excitation (blue) and 488nm excitation (green), respectively. (B) The same root shown in (A) after treatment with 10mM H_2O_2 for ten minutes showing an increase in fluorescence after excitation at 405nm compared to 488nm. (C) Treatment with 10mM dithiothreitol (DTT) for ten minutes restores ratio to untreated WT levels. Treatments were made by gently flushing the distilled water under the coverslip with H_2O_2 or DTT. (D) Bar graph shows the ratio of *mt-roGFP2* in an oxidised versus reduced state for each treatment (N=3). Error bars show standard error of three independent treated seedlings imaged sequentially on the same day. Letters represent significance (adj. $p < 0.05$) using a one-way ANOVA and Tukey's HSD test. Scale bar represents 100 µm. The experiment was repeated with similar results. The seed line used in this experiment was also independently validated[57].

Supplementary Figure S2. Movie of AG2 infected Arabidopsis root stained with aniline blue showing colonisation of AG2 through the cortex into the vascular tissue. Images collected using a Nikon 40x DIC objective.

Supplementary Figure S3. Movie of AG8 infected Arabidopsis root stained with aniline blue showing colonisation of AG8 through the cortex and into the vascular tissue. Images collected using a Nikon 40x DIC objective.

Supplementary Figure S4. A tilescan image capturing the above and below ground tissue of an AG2-1 infected *mt-roGFP2* plant showing extensive foliar necrosis at four days post infection (A). Although the leaf tissue shows significant necrosis, the roots are still expressing GFP through to the primary root tip. The plant roots were stained with propidium iodide, rinsed in water and imaged using a 5x11 tilescan at 25x magnification with a Leica M205FA stereo microscope. (B) Close-up of the rosette shown in (A) compared to the rosette of a mock infected plant (C). Scale bar represents 5cm (A) and 1cm (B-C).

Supplementary Figure S5. Loss of JA, ET and PEN2 mediated defense pathways compromise resistance to AG8. Infection of Col-0, *pen1*, *pen2*, *pen3* single mutants, the *eds1 pen3* double mutant and *pen3 rar1 stg1b* and *coi1 ein2 pen2* triple mutants with AG8. Three independent replicate infections of agar grown plants were performed by transferring 4 plants per biological replicate into mock or *R. solani* AG8 infected pots to produce the data in this figure. Plants were scored for survival at fourteen days post infection at which point surviving plants had started to flower. Letters represents significance (adj. $p < 0.05$) using Fisher's Exact Test. Error bars represent standard error.

Supplementary Table S1. Arabidopsis lines used in this study.

Supplementary Table S2. Primers used to screen mutant lines.

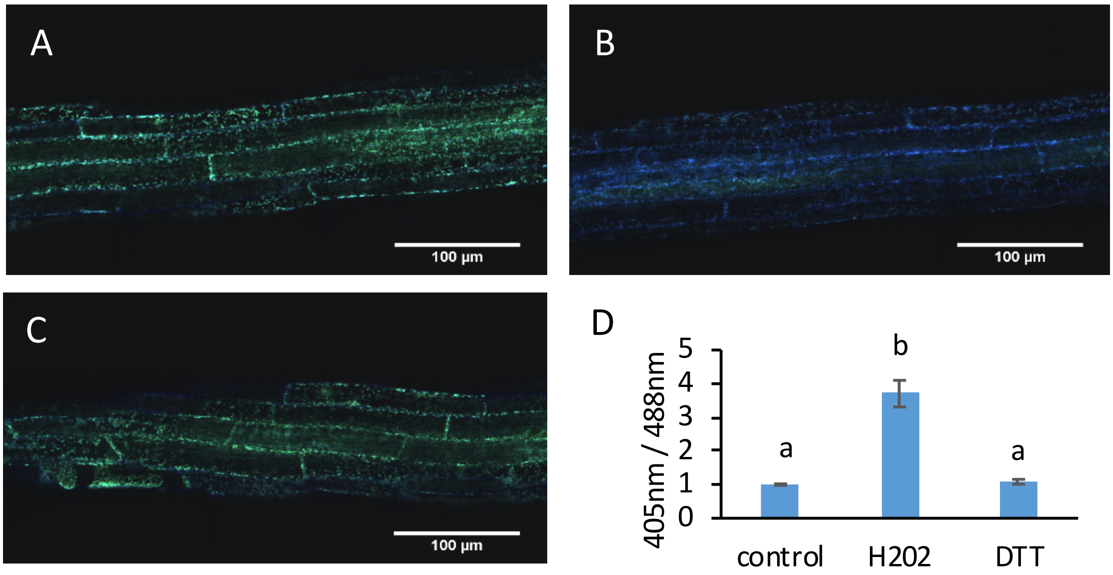
A
100 µm
B
100 µm
C
100 µm
D
405nm / 488nm
5
4
3
2
1
0
a
b
a
control
H202
DTT

Figure S1.

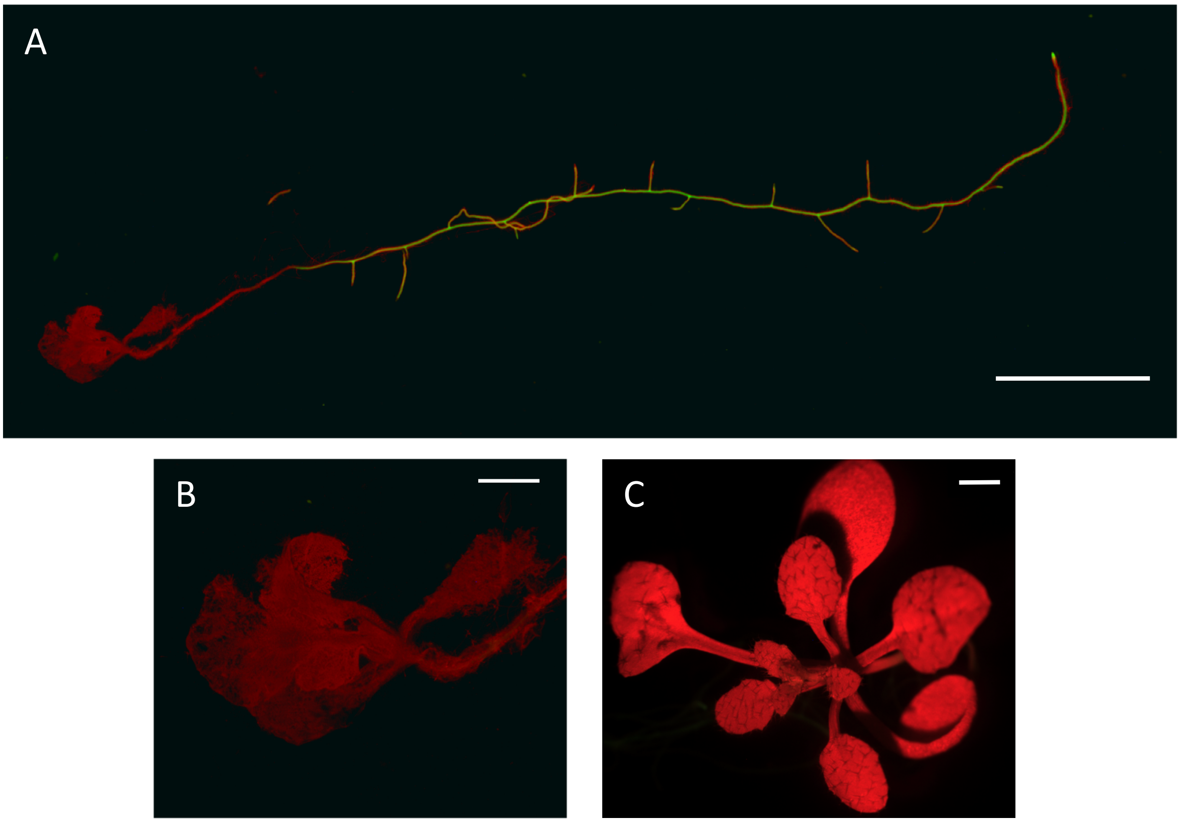

Figure S4.

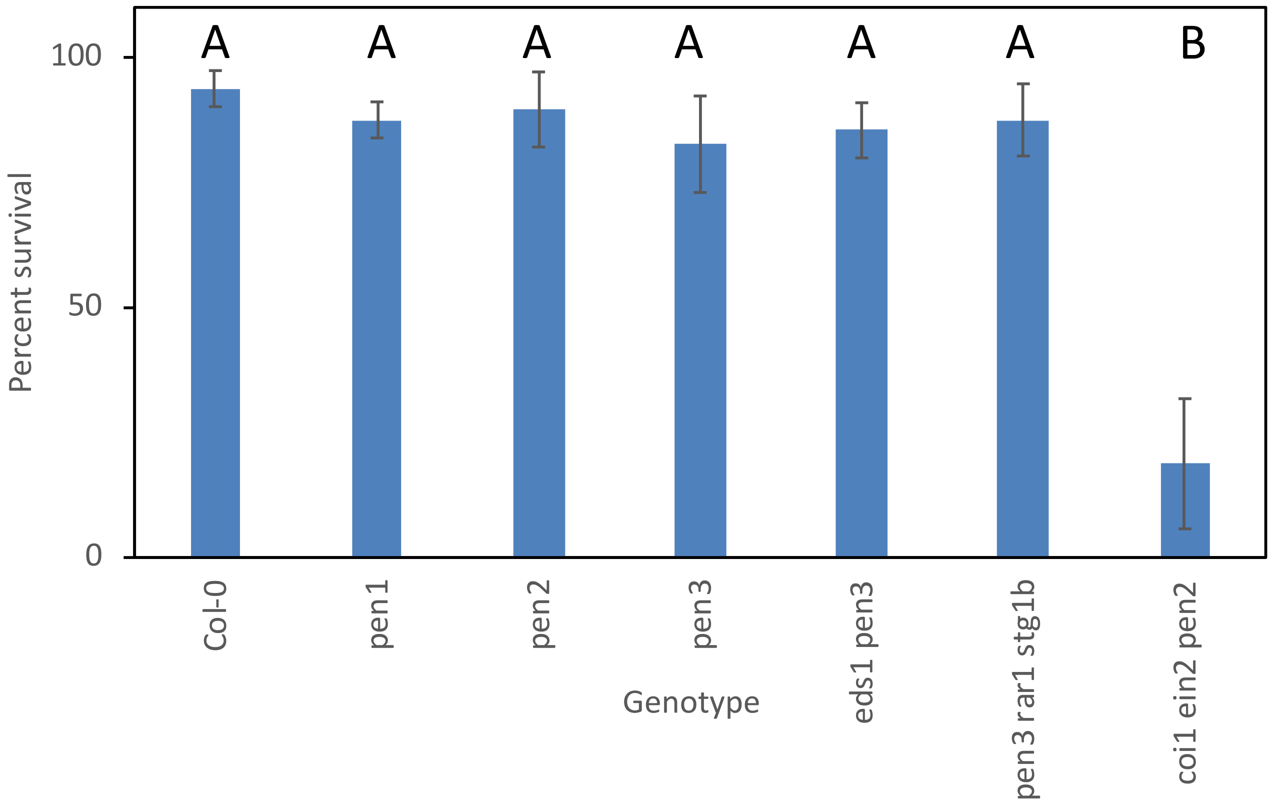

A
A
A
A
A
A
B
100
50
0
Percent survival
Col-0
pen1
pen2
pen3
eds1 pen3
pen3 rar1 stg1b
coi1 ein2 pen2
Genotype

Figure S5.

Supplementary Table S1: Arabidopsis lines used in this study

**Locus**	**Mutant/Transgenic line**	**Stock name / reference**
N/A	*mt-roGFP2*	[1]
AT3G11820	*pen1-4*	CS66945
AT2G44490	*pen2-3*	CS66946
AT1G59870	*pen3-1*	CS66467
AT3G11820, AT1G59870	*pen1-1 pen3-1*	CS67132
AT2G44490, AT1G59870	*pen2-3 pen3-1*	CS67133
AT3G11820, AT2G44490, AT1G59870	*pen1 pen2 pen3*	[2]
AT3G48090, AT1G59870	*eds1-1 pen3-1*	CS67134
AT5G51700, AT4G11260, AT1G59870	*rar1-10 sgt1b-1 pen3-1*	CS67926
AT2G39940, AT5G03280, AT2G44490	*coi1-16 ein2-1 pen2-4*	CS67818
AT2G39940, AT2G40940, AT2G44490	*coi1-16 ers1-1 pen2-4*	CS67821
AT2G39940, AT1G04310, AT2G44490	*coi1-16 ers2-1 pen2-4*	CS67822

References

1 Schwarzländer, M. *et al.* Confocal imaging of glutathione redox potential in living plant cells. *J. Microsc.* **231**, 299-316, (2008).

2 Johansson, O. N. *et al.* Role of the penetration-resistance genes *PEN1*, *PEN2* and *PEN3* in the hypersensitive response and race-specific resistance in *Arabidopsis thaliana*. *Plant J.* **79**, 466-476, (2014).

Supplementary Table S2. Primers used to screen mutant lines

Gene	Mutant	Primer name	Primer sequence	Notes	Reference
PEN1	*pen1-1*	pen1-1F	GAAACACTCTCTTCATGTCACGCG	CAPS - Digestion with MluI	1
		pen1-1R	GAGGACAGAGGTCCTGGTTCG	CAPS - Digestion with MluI	
PEN2	*pen2-1*	pen2-1F	TTTGGAACTGCTTCATCTTCTTATCAGG	CAPS - Digestion with AlwI	1
		pen2-1R	CCTGTACAAGAAATCAATCACAGATCTTCA	CAPS - Digestion with AlwI	
	pen2-4	pen2-4F	AAACGTTGCCGTTGATTTCT	CAPS - Digestion with AciI	2
		pen2-4R	CAGCAACACTAGCGCCATTA	CAPS - Digestion with AciI	
PEN3	*pen3-1*	pen3-1F	TGAAAGCTTCTGCTGCTCAA	CAPS - Digestion with HphI	1
		pen3-1R	TGAGGTGAACGATTTGTTGC	CAPS - Digestion with HphI	
COI1	*coi1-16*	coi1-16 P2	GAACACAATTTAGTACTAAGGACGCATTCCCAA	PCR	3
		coi1-16 P3	AACTAGTTGGGTTCTTTAAGGCTGCAGCTAACT	PCR	
		coi1-16 P4	AACTAGTTGGGTTCTTTAAGGCTGCAGCTATTC	PCR	
EIN2	*ein2-1*	ein2-1F	TGCGAGTAACAGAGCGGAAG	CAPS - Digestion with AflII	4
		ein2-1R	TACATCAGAGTCTTCTTTAAGACTAC	CAPS - Digestion with AflII	

References

1 Johansson, O. N. *et al.* Role of the penetration-resistance genes *PEN1*, *PEN2* and *PEN3* in the hypersensitive response and race-specific resistance in *Arabidopsis thaliana*. *Plant J.* **79**, 466-476, (2014).

2 Westphal, L., Scheel, D. & Rosahl, S. The *coi1-16* mutant harbors a second site mutation rendering PEN2 nonfunctional. *Plant Cell* **20**, 824-826, (2008).

3 Adams, E. & Turner, J. COI1, a jasmonate receptor, is involved in ethylene-induced inhibition of Arabidopsis root growth in the light. *J. Exp. Biol.* **61**, 4373-4386, (2010).

4 Tsuda, K., Sato, M., Stoddard, T., Glazebrook, J. & Katagiri, F. Network properties of robust immunity in plants. *PLoS Genet.* **5**, e1000772, (2009).
